# Quantification of Encapsulated Bioburden in Spacecraft Polymer Materials by Cultivation-Dependent and Molecular Methods

**DOI:** 10.1371/journal.pone.0094265

**Published:** 2014-04-15

**Authors:** Anja Bauermeister, Alexander Mahnert, Anna Auerbach, Alexander Böker, Niwin Flier, Christina Weber, Alexander J. Probst, Christine Moissl-Eichinger, Klaus Haberer

**Affiliations:** 1 Compliance Advice and Services in Microbiology GmbH, Cologne, Germany; 2 Department for Microbiology and Archaea Centre, University of Regensburg, Regensburg, Germany; 3 Lehrstuhl für Makromolekulare Materialien und Oberflächen (Macromolecular Materials and Surfaces), DWI at the RWTH Aachen e.V., RWTH Aachen University, Aachen, Germany; Belgian Nuclear Research Centre SCK/CEN, Belgium

## Abstract

Bioburden encapsulated in spacecraft polymers (such as adhesives and coatings) poses a potential risk to jeopardize scientific exploration of other celestial bodies. This is particularly critical for spacecraft components intended for hard landing. So far, it remained unclear if polymers are indeed a source of microbial contamination. In addition, data with respect to survival of microbes during the embedding/polymerization process are sparse. In this study we developed testing strategies to quantitatively examine encapsulated bioburden in five different polymers used frequently and in large quantities on spaceflight hardware. As quantitative extraction of the bioburden from polymerized (solid) materials did not prove feasible, contaminants were extracted from uncured precursors. Cultivation-based analyses revealed <0.1–2.5 colony forming units (cfu) per cm^3^ polymer, whereas quantitative PCR-based detection of contaminants indicated considerably higher values, despite low DNA extraction efficiency. Results obtained from this approach reflect the most conservative proxy for encapsulated bioburden, as they give the maximum bioburden of the polymers irrespective of any additional physical and chemical stress occurring during polymerization. To address the latter issue, we deployed an embedding model to elucidate and monitor the physiological status of embedded *Bacillus safensis* spores in a cured polymer. Staining approaches using AlexaFluor succinimidyl ester 488 (AF488), propidium monoazide (PMA), CTC (5-cyano-2,3-diotolyl tetrazolium chloride) demonstrated that embedded spores retained integrity, germination and cultivation ability even after polymerization of the adhesive Scotch-Weld 2216 B/A. Using the methods presented here, we were able to estimate the worst case contribution of encapsulated bioburden in different polymers to the bioburden of spacecraft. We demonstrated that spores were not affected by polymerization processes. Besides Planetary Protection considerations, our results could prove useful for the manufacturing of food packaging, pharmacy industry and implant technology.

## Introduction

In course of space exploration, the potential danger to contaminate celestial bodies with terrestrial microorganisms (or *vice versa*) has received growing attention. The goal of planetary protection is to mitigate such risks, which are most severe in case of missions to planets where conditions are favorable for life. In order to reduce the risk of contamination and ensure the scientific value of possible life detection missions, the Committee on Space Research (COSPAR) has issued specific regulations for spacecraft cleanliness [1]. Spacecraft under planetary protection limitations are constructed in clean rooms and maintained under bioburden control to diminish risks of microbial contamination. Sampling of the spacecraft-associated microbiome (including microbial heat shock resistant bioburden) is employed to study the microbial diversity of the cleanroom environment, spacecraft elements, and the assembled spacecraft (e.g. [2], [3], [4], [5], [6], [7], [8]). Airborne contamination is collected by active sampling (the most commonly used samplers in the pharmaceutical and medical device industry are impaction and centrifugal samplers [9]), while bioburden on surfaces can be detected by the use of swabs, wipes, or similar devices [10], [11], [12]. However, these sampling procedures cannot cover all sources for microbiological contamination. A potential contamination pathway that escapes such standard controls is the bioburden encapsulated in polymers, which are widely applied in the construction of spacecraft [13].

For modern spacecraft hardware lighter but often more porous composite materials are preferred over those heavy stainless steel materials used in the Viking era. Beside metal-based materials, spacecraft hardware comprises silicones (e.g. in electronic components, optics, and solar cells), other polymeric materials (e.g. parachutes, airbags, thermal covers, coatings, paints, and wires) and carbon-polyesters used for structures. Due to their porous structure these new materials increase the risk of an accidental contamination of extraterrestrial environments [14].

Bacterial spores embedded or encapsulated in these pores are – to a certain degree – protected from stresses like space vacuum and radiation during space travel and from heating during entry into a planet’s atmosphere [15], [16], [17], [18], [19], [20], [21], [22], [23]. Such sheltered bioburden has to be considered, since materials degrade, and some parts of a spacecraft intended to land on a planet’s surface may break (e.g. the head shield, the back shell, parts of the active descent system such as engines). In case such impacts do not result in complete sterilization of spacecraft hardware due to entry heating, they may have some potential to inoculate microbes into favorable environments of a planet’s bottomset beds [24], [25].

The risk of bioburden enclosure in polymers is not equal for all materials. Structural materials like polyimides are cured at high temperature conditions of several hundred °C, which are not survived even by the most resistant spore-forming microorganisms known. Other polymers are used in spacecraft parts which are treated under very stringent conditions, e.g. the heat shield, which is degassed for extended periods at elevated temperatures. Some polymers are cured from organic precursors, which are microbicidal to vegetative microorganisms but not spores. Some, polymers contain fillers or strengthening enclosures that may carry a different microflora than the encapsulating polymer itself and may protect embedded bioburden.

For the purpose of this study, “encapsulated bioburden” was defined as heat-shock resistant bioburden inside (i.e. not free for gas/water vapor exchange) non-metallic materials. This includes microorganisms physically surrounded by and in direct contact with the polymer matrix, and those indirectly surrounded by polymer in pores, bubbles or protected by non-polymeric materials (e.g. fillers, insulating or reinforcing structural components).

Detection of encapsulated microorganisms in cured polymers is a challenging task, because harsh and destructive extraction methods are needed to release microorganisms from these materials. In a recent study [26] *Bacillus pumilus* SAFR-032 spores were artificially encapsulated in poly(methylmethacrylate) (Lucite, Plexiglas) and released by dissolution in organic solvents. However, for most other polymers no effective solvents are available. In the present communication, we used Scotch-Weld 2216 B/A adhesive as a model polymer to determine the survivability of purposely encapsulated microorganisms, to investigate the effectiveness of spore recovery from uncured precursor, and to estimate the intrinsic bioburden in polymers. The aim of this study was to develop cultivation- and molecular-based methods for assessing the microbial bioburden within polymeric materials and thus be able to estimate their quantitative contribution to the total bioburden of spacecraft.

## Materials and Methods

### Bacterial Strains and Cultivation Conditions

Bacterial strains were purchased from the Leibniz Institute DSMZ – German Collection of Microorganisms and Cell Cultures (Braunschweig, Germany). *Bacillus safensis* DSM 19292^T^ was selected as the standard spore model for most experiments due to its high resistance to various environmental stresses and as an obvious contamination source of spacecraft and clean room environments (*B. safensis* was isolated from the surface of the Mars Odyssey Orbiter spacecraft and also the assembly-facility surfaces at Jet Propulsion Laboratory and Kennedy Space Center [27]). Available spore preparations revealed a high number of germinable spores, (17.6%) in contrast to *Geobacillus stearothermophilus* (<5%) used only for visualization of spores in the embedding model (see below). Hence, the number of viable but non-germinable spores (VBNG) was kept low. Spore suspensions were prepared by lysozyme (200 ng/µL) and DNase treatment followed by heat shock (15 min, 80°C) as described previously [28], [29]. For the experiments, *B. safensis* was incubated for 2–5 d at 32°C on soybean casein digest agar (Becton Dickinson, Heidelberg, Germany). Spores of *Geobacillus stearothermophilus* DSM 5934^T^ were collected from agar medium and incubated in 65% 2-propanol for 3 h (at room temperature), followed by two wash cycles with deionized water. *G. stearothermophilus* was incubated 2–5 d at 55–60°C on soybean casein digest agar.

### Polymer Materials

The analyzed materials for flight hardware construction and their preparation are listed in **Table 1**. For each material, the most suitable solvent for dissolution or dilution of the uncured polymer was determined by mixing precursors with different solvents (at concentrations of 10–50 wt.-%) and vigorous vortexing. The samples were incubated up to 24 h at room temperature to observe if sedimentation or polymerization occurred.

**Table 1:** List of materials analyzed, their preparation, and the solvent most suitable for dilution of the uncured precursors.

Name (Supplier)	Type	Utilizationin spacecraft	Preparation	Solvent usedfor extraction	Density (g/cm^3^)
Scotch-Weld 2216 B/A (3M, France)	Epoxy adhesive	screw locks, bonding	component A (hardener): component B (base) = 7∶5 (w/w)^1^	2-propanol	1.32
SG121FD (MAP, France)	Silicone coating	Thermal control paint	base : catalyst = 86∶14 (w/w)	MAP SG121FD thinner	1.41
Solithane 113 (Specialty Polymers & Services, USA)	Urethane resin	conformal coating, screw locks, bonding, insulation	S113 : C113–130 (hardener) = 100∶70 (w/w)^2^	ethanol	1.07
ESP 495 (ACC Silicones/EADS Astrium)	Silicone adhesive	Adhesive for thermal protection system	–	acetone	1.07
Dow Corning 93–500 (Ellsworth Adhesives GmbH, Germany)	Silicone encapsulant	Sealing and bonding	encapsulant : curing agent = 10∶1 (w/w)^2^	acetone	1.08

1 mixed thoroughly for 5 min.

2 mixed thoroughly for 1–2 min.

### Surface-embedding of Spores in Scotch-weld 2216 B/A

*B. safensis* or *G. stearothermophilus* spores were washed twice in acetone and diluted to obtain an acetonic spore suspension of ∼1×10^5^ colony forming units (cfu)/mL. One drop of the suspension was spread on the surface of water-soluble polymer (poly vinyl alcohol (PVA)) and dried for 2 d at room temperature. 1–2 g of freshly prepared Scotch-Weld 2216 B/A (**Table** **1**) were placed on top of the dried spore suspension on PVA. The polymer was allowed to cure for 7 d at room temperature. The water soluble PVA was peeled off or washed from the sample after curing, leaving embedded spores concentrated in a layer proximal to the surface of the material.

### Scanning Electron Microscopy (SEM) and Dual Beam Focused Ion Beam (FIB)

**Figure 1:**
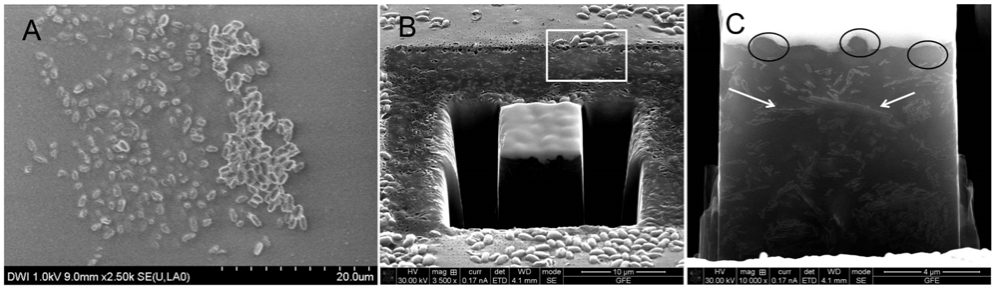
Surface embedded spores of *G. stearothermophilus* in cured Scotch-Weld 2216 B/A. (A) Scanning electron micrograph. Spores on the right side of the image appear sharply outlined and are apparently either surface-attached or partially embedded and protruding from the surface of Scotch-Weld 2216 B/A. Spores on the left side of the image appear more deeply embedded below the surface. (B) Overview of embedded spores at starting position of dual beam FIB/SEM measurement. The rectangle shows the area where the FIB ablation was performed to image the sample cross section. Spores partially embedded in the surface of cured Scotch-Weld 2216 B/A are visible. During sectioning of the samples with FIB, which removes layer after layer of 1 µm thickness each, vertical sections can be visualized by SEM to obtain a side view of the embedded spores in the adhesive (Fig. 1, C). (C) The outline of spores embedded in or near the surface of Scotch-Weld 2216 B/A can be seen along the cross section. Spores are partially or fully embedded (circles). Kaolin (filler material in Scotch-Weld 2216 B/A) inclusions are also visible (marked by arrows).

Samples of inoculated surfaces of the adhesive Scotch-Weld 2216 B/A were prepared on a dual-beam FIB/SEM workstation (FEI Helios Nanolab) and investigated by field emission scanning electron microscopy (FESEM) using a Hitachi SU-4800 instrument operated at 1 kV. Prior to inserting the sample in the FIB, a thin Au layer was sputter-deposited on the surface of the sample to protect the polymer in the subsequent preparation steps. FESEM scanned the specimen in a grid to create an image. FIB cut out a 10 µm by 10 µm test area with an ion beam perpendicular to the specimen surface, which was then sectioned by the ion beam in 1 µm steps. Images were taken by an SEM vertically oriented to the ion beam. FESEM images of PVA, and inoculated and non-inoculated Scotch-Weld 2216 B/A allowed visualization of embedded spores in the adhesive. FIB/SEM images verified the partial embedding of the spores necessary for further staining approaches (see **Fig. 1**).

### Fluorescence-based Detection of Surface-embedded Spores and Confirmation of their Identity after Re-growth

Detection of (partially) embedded, intact spores in Scotch-Weld 2216 B/A was performed by staining spores with AlexaFluor succinimidyl ester 488 (AF488, MoBiTec, Göttingen, Germany) prior to the embedding procedure and with propidium monoazide (PMA, Biotium, Hayward, CA, USA) subsequent to embedding. AF488 is a fluorescent dye that can be applied for the visualization of many vegetative cells [30] as well as spores [29]. For labeling, spores were exposed to 100 µl ATTO-dye labeling buffer (ATTO-TEC GmbH, Siegen, Germany), to yield unprotonated amino groups of proteins, and 10 µg AF488 were added. Reaction between dye and proteins was enabled at RT in the dark on a shaker for 30 min. Stained spores were washed three times with sterile water to remove unspecific staining. PMA selectively enters wall-compromised cells and binds to DNA [31] as has also been demonstrated for heat-inactivated *B. safensis* spores [29]. After treatment of spores with 10 mM dithiothreitol (DTT) at 65°C for 15 min (to increase permeability), they were stained with 50 µM PMA in the dark for 50 min. Cross-linking of PMA with DNA was enabled via halogen light (500 W) exposure for 3 min of samples kept on ice. The labeling procedure was completed by three serial washing steps. Cy5 (cyanine dye 5) was applied as a surface marker, by adding 2 µL of an aqueous dye stock solution (30 µM) on top of the spore preparation.

50 mM CTC (5-cyano-2,3-diotolyl tetrazolium chloride, Polysciences Europe GmbH, Eppelheim, Germany) was used to visualize metabolic activity of *B. safensis* during its germination process. Spore germination was provoked by incubation in growth medium or glucose solution (at 32°C, approx. 1–2 h; either in tubes or for embedding models covered with growth media and incubated horizontally). Slightly attached and not properly embedded spores were removed by sonication at maximum intensity (120 W, 35 kHz) for 10 min in a 50 mL falcon tube filled with 35 mL of sterile distilled water to guarantee evaluation of embedded or encapsulated spores. Fluorescently labeled spores were monitored via confocal laser scanning microscopy (CLSM) as described previously [29], using an inverse Laser Scanning Microscope (LSM 510-Meta confocal microscope, CLSM, Zeiss, Munich, Germany). Fluorescence signals of PMA or CTC (excitation 514 nm, detection 505 nm), AF488 (excitation 488 nm, detection 505–530 nm) and Cy5 (excitation 633 nm, detection 650 nm) were detected in the multi-track mode to avoid cross-talk phenomena. Z-stacks were scanned with a 50% overlap of each 0.4 µm section. CLSM images were analyzed, arranged and 3D projections were made with the Zeiss LSM Image Browser Software.

CLSM monitored embedding models were subjected to cultivation dependent analysis to support conclusions drawn from fluorescence staining of embedded spores. Scotch-Weld 2216 B/A surfaces were cleaned with 70% (v/v) ethanol to minimize contaminations caused by non-sterile CLSM procedures. For cultivation 1 mL tryptic soy agar was poured on model surfaces and incubated at 32°C for 4 d. Grown colonies were identified (Geneart Life Technologies, Regensburg, Germany) by their PCR amplified *rrnB-16S* and *gyrB* gene sequences (as *B. safensis* and *B. pumilus* show 99.9% identity on *rrnB-16S* gene level but show adequate differences (91.2% identity) in their respective *gyrB* genes [27]. Bacteria-specific primers 9 bF (GRGATCCTGGCTCAG) [32] and 1406uR (ACGGGCGGTGTGTRCAA) [33] (1.25 ng/µL each) were used for *rrnB-16S* gene PCR containing 200 µM dNTP mix, 2 U of *Taq* DNA polymerase in Buffer Y with MgCl_2_ (10x). DNA templates were amplified in 10 cycles of denaturation at 96°C for 30 sec, annealing at 60°C 30 sec, elongation at 72°C for 1 min, followed by 25 cycles of a changed denaturation at 94°C for 20 sec. For *gyrB* gene targeted PCR 1 µg of template DNA was amplified with UP-1long (GAAGTCATCATGACCGTTCTGCA(TC)GC(TCAG)GG(TCAG)GG(TCAG)AA(AG)TT(TC)AG) and UP-2r (AGCAGGGTACGGATGTGCGAGCC(AG)TC(TCAG)AC(AG)TC(TCAG)GC(AG)TC(TCAG)GTCAT) primers [34], [35] (1 µM each). PCR settings were adjusted to the following conditions: 30 cycles of denaturation at 94°C for 1 min, annealing at 58°C 1.5 min, and elongation at 72°C for 2.5 min. PCR sequences were compared with GenBank deposited sequences (http://www.ncbi.nlm.nih.gov/genbank/) using the basic local alignment search tool (blastn; http://blast.ncbi.nlm.nih.gov/Blast.cgi) [36].

### Survival of *B. Safensis* Spores in Uncured Polymer Precursors

To determine the possible sporicidal effects of the uncured polymer precursors, a *B. safensis* spore suspension (10^7^ cfu/mL) was washed twice in its respective solvent (depending on the material tested, see **Table 1**). 0.5 g of freshly-prepared polymer was mixed with the final spore pellet and incubated 0–60 min at room temperature. After adding 1 mL of solvent to the polymer-spore mixture and vigorous mixing, the resulting suspension was serially diluted in sodium chloride peptone buffer. The appropriate dilutions were subjected to heat shock (80°C, 10 min, according to specifications of US Pharmacopeia [37]) and pour-plated on soybean casein digest agar. For comparison the cultivable spore number after washing and resuspending in solvent without polymer was determined. For these experiments, growth conditions were always identical for experimental and control samples. It was not attempted to optimize recovery conditions for *B. safensis*, as this organism served as model to comparatively evaluate the effect on spore survival of the polymer precursors. Recovery of viable spores after incubation was always lower than the inoculum, which is to be expected due to losses during the experimental procedures, but generally high enough to reasonably expect spores to survive in polymer precursors.

### Recovery of *B. Safensis* Spores Grown in the Presence of Polymer Precursors on Nutrient Agar

Components of the polymer materials were prepared as shown in **Table 1**, diluted in the appropriate solvent (0.1–0.5 g/mL, depending on the solubility of the precursors), and inoculated with *B. safensis* spores to reach a final spore concentration of 10^2^ cfu/mL. *B. safensis* spores (10^2^ cfu/mL) in solvent only (without the polymer precursors) served as a positive control. Samples were subjected to heat shock (80°C, 10 min) to activate endospores. 1 mL aliquots were pour-plated in soybean casein digest agar. Plates were incubated 5 d at 30–35°C to detect inoculated spore-formers and to check if residues of the polymer precursors inhibited germination and growth. All colonies appeared within 2 days, no additional growth was observed when incubation was prolonged. All our solvent extraction methods achieved recovery rates above 10% of the positive controls and were thus considered suitable.

### Cultivation-based Recovery of Intrinsic Endospore Burden from Polymer Precursors

Components of the polymer materials were prepared as shown in **Table 1**, and diluted in the appropriate solvent (0.1–0.5 g/mL, depending on the solubility of the precursors). The method was optimized for detection of cultivable bacterial endospores by including a heat activation step before pour-plating 1 mL aliquots on soybean casein digest agar or R2A agar (Sigma-Aldrich; Steinheim, Germany). Plates were incubated 5–7 d at 30–35°C, followed by 5–7 d at 55–60°C to detect mesophilic and thermophilic spore-formers, respectively. In total, 45–80 cm^3^ of each material was investigated. If colonies were detected, they were isolated and visualized microscopically after Gram-staining.

### Calculation of the Encapsulated Bioburden in Precursors Corrected for the Recovery Efficiency

To calculate the corrected value for mean encapsulated bioburden (CME), the recovery efficiency was included in the determined colony number.

Recovery efficiency (RE) was calculated by
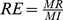
with a standard deviation of 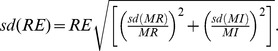


Here: MR = mean recovered spores from the polymer precursors, MI = mean inoculated spores, and calculated standard deviations sd(MI) and sd(MR).

Tests for bioburden were repeated 4–10 times for each material, yielding mean and standard deviation of the number of colonies recovered from a certain volume of material (ME, sd(ME).

Mean encapsulated bioburden 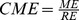, with standard deviation:
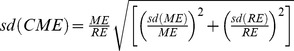
The values given in this report are conservative values, calculated by 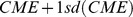. When 0 cfu were recovered, calculations were performed with 1 cfu, and CME was given as less than (<) the resulting value.

### Cultivation-based Determination of Bioburden in Kaolin

To determine the bioburden of kaolin (filler material of Scotch-Weld 2216 B/A), samples of kaolin in 3 grain sizes (ASP 200, 600, and 900) were obtained from a supplier in Germany (Quarzwerke, Frechen, Germany). Of each grain size, 10 g were suspended in duplicate in 90 mL sodium chloride peptone buffer (Merck kGaA, Darmstadt, Germany, prepared according to manufacturer’s instructions) and subjected to heat shock (80°C, 10 min) or incubation at room temperature in parallel. Of each sample, 0.1 mL in 10 replicates was spread-plated on R2A agar, and plates were incubated for 7 d at 30–35°C. The respective contribution of kaolin to the weight of the adhesive was taken into account (8.9% for ASP 200, and 11.4% for ASP 600 and ASP 900 each) to determine the total bioburden of Scotch-Weld 2216 B/A.

### DNA Extraction from Kaolin and Purification

DNA was extracted from kaolin (filler in Scotch-Weld 2216 B/A) with the Precellys Bacterial/Fungal DNA kit (Peqlab, Erlangen, Germany). 0.1 g kaolin (of three grain sizes: ASP 200, 600 and ASP 900; in triplicates) was suspended in 200 µL Tris-EDTA (TE) buffer. Suspensions were transferred to a 2 mL Precellys tube and processed as indicated by the manufacturer. The extracted DNA (solved in 50 µL Elution Buffer) was purified by precipitation overnight at −20°C with the same volume of ice-cold 2-propranol (abs.), followed by washing with ice-cold ethanol (70% v/v) and drying of the pellet for 3 h. DNA was resuspended in 15 µL PCR-grade water and stored at −20°C till further use. DNA-free extraction blanks for qPCR experiments were prepared by suspension of a mixture of ASP 200, ASP 600 and ASP 900 (0.033 g each, triplicates) in 1 mL of sterile DNaseI buffer (100 mM Tris-HCl; pH 7–7.5; 25 mM MgCl_2_; 5 mM CaCl_2_). 2 µL of DNaseI (1 mg/mL) were added to the kaolin suspension to digest present DNA by incubation at 37°C for 1 h. The enzyme was inactivated at 90°C for 1 h, and removed by washing three times with sterile water. After 2 d of drying at room temperature, the extraction blanks were subjected to the DNA extraction and purification procedure as described above.

### DNA Extraction from Polymer Precursors

Samples were prepared by dissolving 5 g Solithane 113 in 10 mL ethanol (abs.). Ethanol inoculated with *B. safensis* spores (2×10^6^) was used as a positive control, and the same volume of ethanol was processed as extraction negative control. Samples were concentrated by centrifugation (8,600×g, 1 min) and resuspension of the pellet in 100 µL TE buffer; however, the formed precipitates remained undissolvable. For decoating of spores, 500 µL decoating buffer (50 mM Tris Base (pH>9.5), 1% (w/v) sodium docecyl sulfate (SDS), 8 M urea, 50 mM dithiothreitol (DTT), 10 mM Na_2_EDTA) were added, followed by incubation at 60°C for 90 min with shaking [38]. Samples were washed three times with Sodium chloride-Tris-EDTA (STE) buffer (10 mM Tris-HCl (pH 8), 10 mM Na_2_EDTA, 150 mM NaCl) and once with Lysis buffer (Precellys Bacterial/Fungal DNA kit, Peqlab). Afterwards, pellets were resuspended in 300 µL Lysis buffer and stored overnight at −20°C. After homogenization of the samples and degradation of cells, following the manufacturer’s instructions as described above (see DNA extraction from kaolin), the suspensions were transferred to peqGold PhaseTrap columns (Peqlab) after short centrifugation (12,000×g, 20–30 sec). The same volume of 24∶25∶1 (v:v:v) chloroform:phenol:isoamyl-alcohol was added and carefully mixed, followed by centrifugation at 1,400×g rpm, 5 min, at room temperature. Subsequently, the supernatant was transferred into a new tube and mixed with 0.057 vol. 7 M ammonium-acetate and 1 vol. of ice-cold 2-propanol (p.a.). For precipitation, the mixture was stored at −20°C ≥ 4 h before washing with 1 mL 70% (v/v) ethanol (16,900 rpm, 30 min, 4°C). The supernatant was discarded and the pellet was dissolved in 15 µL water after 1 h drying at room temperature.

### Quantitative PCR (qPCR)

Reaction mix for each sample (final volume 20 µL) consisted of 10 µL SYBR Green mix (Qiagen, Hilden, Germany), 1 µL forward (338 bF, ACTCCTACGGGAGGCAGCAG) and reverse primer (517 uR, GWATTACCGCGGCKGCTG; 25 ng/µL each)) [33], [39], 7 µL H_2_O and 1 µL template (DNA sample or extraction blank or water (negative control)). Prepared qPCR suspensions were gently mixed and tubes were loaded into the Rotor Gene 6000 Cycler. The PCR program was adapted from the SYBR Green protocol (Qiagen) with a hot start step for polymerase activation at 95°C for 15 min and 40 cycles of 94°C for 15 sec, 60°C 30 sec, 72°C 30 sec. qPCR amplification was terminated with a melt curve from 72°C to 95°C. For analysis of the amplification results, the Rotor Genes 6000 software was used. 1 µL of the *rrnB-16S* gene PCR product of *Bacillus atrophaeus* was included as a standard (10^4^ to 10^6^ *rrnB-16S* gene copies). Since DNA templates extracted from kaolin interfered with the qPCR, one standard dilution was mixed with a DNA-free extraction blank from kaolin in order estimate the effect on the amplification process. The inhibitory effect in kaolin samples was calculated according to differences between the kaolin extraction blank + standard and the pure standard. This value was used to correct kaolin sample values without an inhibiting effect. Values were equalized to 1 g kaolin and the estimated copy numbers were divided by a mean of all known bacterial *rrnB-16S* gene copy numbers per cell (4.1 *rrnB-16S* gene copies per cell; source: ribosomal database rrnDB [40], [41]).

## Results

### General Remarks and Test Strategy

The test strategy to determine encapsulated bioburden was developed with Scotch-Weld 2216 B/A as an example polymer, because it combined several relevant features. The physical structure of the cured polymer was found suitable for electron and epifluorescence microscopy, and it contained an additional component of filler material, which needed to be investigated. The precursors of the polymer could be dissolved in 2-propanol.

Two test models were developed and afterwards applied to different polymers when feasible.

### Determination of Structural Integrity and Viability of Encapsulated Endospores in Cured Polymer (Test Model I)

A surface embedding model was newly developed as a method to allow easy localization of intentionally embedded spores in the cured polymer block, and to facilitate the access for microscopic and electron microscopic techniques. Inoculated spores were partially or completely embedded to a depth of several spore diameters. Hence, they were directly accessible for analytical examination without the necessity of sectioning or fractioning to disrupt the polymer. This preparation method allowed application of a range of detection methods as shown in Results Section 2. This test model was only applied to Scotch-Weld 2216 B/A.

### Investigation of Uncured Polymer Precursors to Quantitatively Estimate the Worst Case Bioburden (Test Model II)

While Test Model I allowed localization and characterization of experimentally embedded spores in high numbers, any inherent bioburden of the polymers would be too dispersed to allow detection by microscopic methods *in situ*. Chemical or physical disintegration of the polymers would have to be applied to extract intrinsic encapsulated microorganisms from cured polymers. In contrast to the experiments performed on lucite [26], solvent dissolution of the cured spacecraft relevant polymers investigated here, was not feasible. Mechanical degradation of the polymer was considered possible, but exceedingly difficult to control with regard to uniformity of the procedure and mechanical spore disintegration. Hence, no advantage was seen in milling or grinding procedures. As an innovative approach, determination of the bioburden of the uncured polymer precursors was pursued. During the terminal step of polymerization (curing) an increase in polymer bioburden is obviously impossible, while physical and chemical processes during polymerization might even reduce viable microorganisms. Hence, bioburden in uncured polymer was considered the most conservative proxy for encapsulated bioburden in cured polymer. As all uncured polymer precursors were found to be soluble in suitable organic solvents, the test model was applicable to all the spacecraft relevant polymers investigated here.

For all uncured polymers, survival of inoculated spores of the *B. safensis* model organism during a 60 min exposure period was verified. This was performed at high spore concentrations (10^7^ cfu/mL) to allow dilution in buffer and to minimize the effect of solvent and polymer residues in the plating assay.

To achieve maximum sensitivity in determination of inherent bioburden, polymer precursors were diluted in solvent as little as possible and pour-plated in agar without transfer to an aqueous diluent. For all investigated polymers germination and colony growth of inoculated spores (*B. safensis* 10^2^ cfu/mL) in presence of residual polymer and solvent was verified. This also allowed determination of the recovery efficiency for the model organism for each given material and method. Recovery results were used to specify the amount of un-inoculated material that had to be extracted to generate significant figures for estimation of intrinsic endospore burden per cm^3^ of cured polymer.

### Application of Test Model I: Detection and Viability Determination of Surface-embedded Spores in Cured Scotch-Weld 2216 B/A

Preliminary experiments had shown that ultra-thin sectioning using a microtome followed by transmission electron microscopy (TEM) was not feasible for the material due to the spongy structure of Scotch-Weld 2216 B/A. Instead, surface-embedding followed by scanning electron microscopy was found to be the method of choice to qualitatively demonstrate the presence and accessibility of embedded spores in Scotch-Weld 2216 B/A. Polymerization of the adhesive Scotch-Weld 2216 B/A on a layer of spores on the inoculated surface of water-soluble poly vinyl alcohol (PVA) yielded a surface with fully embedded, partially embedded and loosely attached spores as shown in **Fig. 1A**. To visualize the effect of encapsulation and polymerization on structure and integrity of *G. stearothermophilus* and *B. safensis* spores, scanning electron microscopy (SEM) in combination with focused ion beam (FIB) electron microscopy were employed.

SEM images of the sample surface confirmed the embedding status (**Fig. 1**). Non-inoculated surfaces were smooth (as seen in in **Fig. 1A** to the right of the embedded spores) without spore structures. Double beam FIB/SEM analysis confirmed the varied depth of surface-embedding as indicated in the SEM analysis. Spores embedded to the depth of several spore diameters could be visualized (**Fig. 1 C**). The double beam FIB/SEM images also showed the presence of filler material in the polymer, which obstructed the clear outlines of the faint shapes of embedded spores.

While the electron-microscopic study allowed visualization of spores and their arrangement with respect to the polymer matrix, for viability determination physiological tests had to be performed. Samples of Scotch-Weld 2216 B/A with surface-embedded *B. safensis* spores were overlaid with nutrient agar after cleaning the surface with 70% ethanol to remove loosely attached spores and inactivate vegetative contaminants. Several colonies developed in the agar, indicating that (partially) embedded spores of *B. safensis* were still cultivable after the curing process.

Application of standard cultivation technique and physiological fluorescent dye staining followed by fluorescence and confocal laser scanning microscopy to surface-embedded samples clearly illustrated the high potential of this preparation method for analysis of polymer-embedded spores.

**Figure 2:**
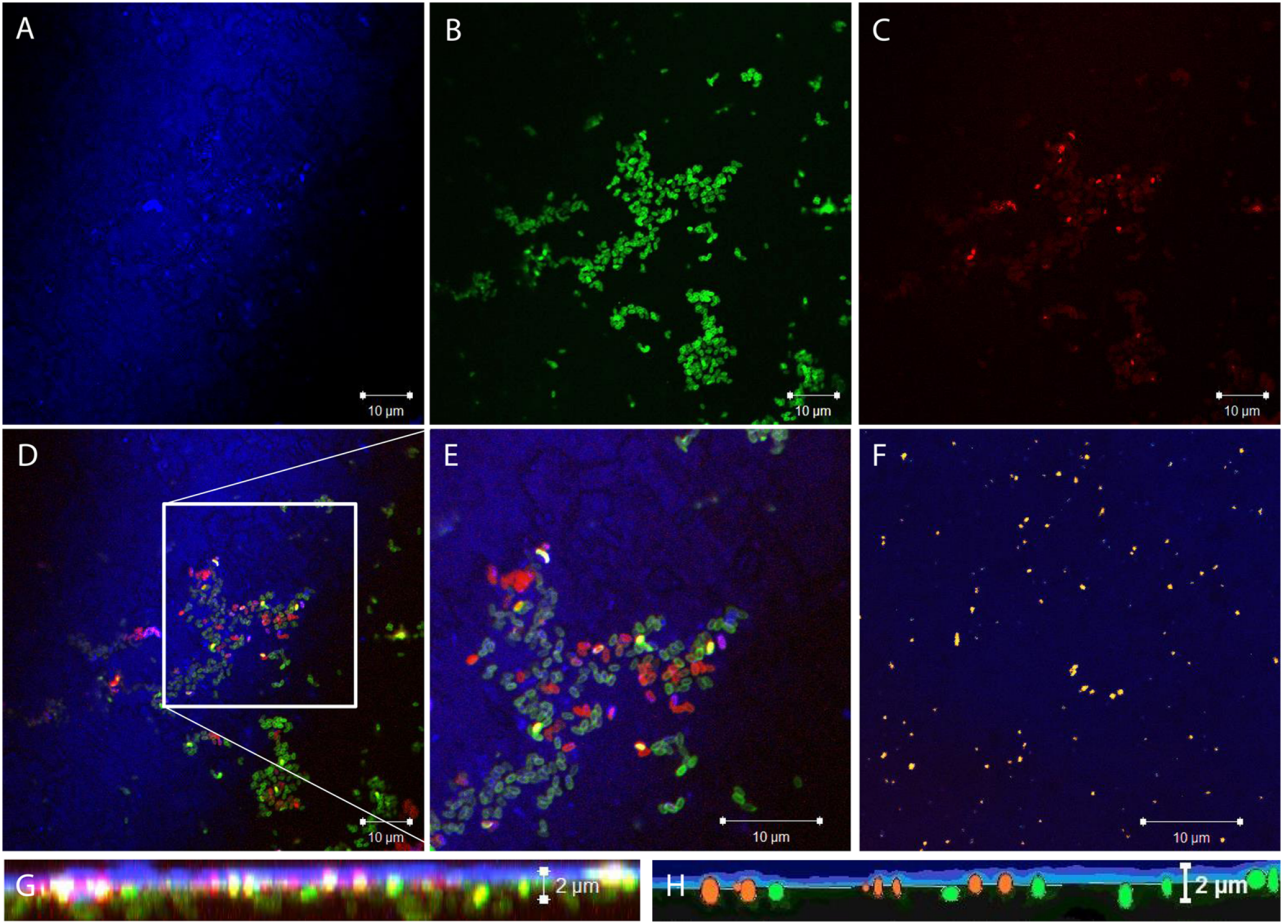
CLSM images of Test Model I. (A) Cy5 channel (blue) to visualize the polymer surface. (B) AF488 channel (green) to distinguish spores from background signals (potential polymer autofluorescence and debris). (C) PMA channel (red) to detect disintegrated spores. (D) overlay of all channels. (E) shows enlarged detail of Fig. 2D. (F) CTC channel (yellow) show spores with metabolic activity in the presence of provided nutrients (different position on Test Model I is shown). (G) z-axe projection from 90° calculated from z-stacks scanned with a 50% overlap of each 0.4 µm section (blue horizontal line indicates the surface of the adhesive). (H) abstracted scheme of Test Model I interpreted from Fig. 2G – intact spores colored in green, disintegrated spores are red-orange, blue glowing line demonstrates polymer surface, gray area represents polymer material. Spores of *B. safensis*, were stained with AF488 prior to embedding (Fig. 2B), but with PMA after embedding (Fig. 2C). The PMA signals were visible in a layer of about 3 µm, whereas the AF488 signal was detectable to a depth of 10 µm (spores were introduced into deeper layers during embedding; PMA could penetrate the polymer to stain spores up to a depth of 3 µm). All images, overlays and projections were achieved with the Zeiss LSM Image Browser Software.

In order to systematically evaluate the depth to which spores could be introduced into the adhesive by the embedding procedure, spores were stained with AF488 prior to embedding. As a surface marker, Cy5 was employed. Full Cy5 fluorescence was present above the adhesive surface in the liquid layer and disappeared on and below the surface. This allowed exact localization of partially embedded spores in Scotch-Weld 2216 B/A (**Fig. 2A**). Controls without embedded spores and stained with Cy5 and AF488 afterwards revealed only scattered locations of AF488 signals, which could be clearly distinguished from spore shapes and spore label intensity. Staining quality and CLSM channel configurations were adjusted and judged for labelled spores in suspension, labelled spores on polymer surface, as well as embedded and encapsulated labelled spores. A slight decrease in label fluorescence could be detected for embedded and encapsulated spores. AF488-prestained spores were successfully detected to a depth of 9.2 µm by CLSM (**Fig. 2B**). In addition, PMA-staining after the embedding procedure was performed as described in [29] to analyze the effect of polymerization on spore integrity *in situ*. Spores could be stained even after being (partially and fully) embedded in Scotch-Weld 2216 B/A in a layer of approximately 3 µm from the surface (**Fig. 2C–E**). Notably, the process of embedding did not significantly change the percentage of PMA-stainable (disrupted) spores compared to the non-embedded control, indicating that most spores were unaffected by the embedding process (**Table 2**). This was confirmed by successful CTC staining, indicating an active oxidative metabolism of (partially) embedded spores of *B. safensis* after reactivation by nutrient supplement (**Fig. 2F**). Colonies that appeared after covering half-embedded spores with nutrient agar were *B. safensis* colonies, as revealed by *rrnB-16S* (99.9% identity) and *gyrB* gene sequencing (96.48% identity to type strain deposited sequences in GenBank: http://www.ncbi.nlm.nih.gov/genbank/).

**Table 2:** Influence of embedding in polymerized Scotch-Weld 2216 B/A on *B. safensis* spore integrity.

Sample	PMA positives (%)	Ratio (spore number)	Signal depth AF488 (µm)	Signal depth PMA (µm)
spore suspension (AF488 + PMA)	2.91	37/1272	-	-
Non-embedded control (AF488 + PMA before embedding)	3,74	52/1390	2.8	2.8
Embedded viable spores (AF488 before, PMA after embedding)	3.29	137/4166	2.8	0.8

Only partially-embedded spores were counted (directly on the surface of Scotch-Weld 2216 B/A, treated by ultrasonication; surface location was confirmed by using Cy5 surface marker).

### Application of Test Model II for Scotch-Weld 2216 B/A: Survival of *B. safensis* Spores in Polymer Precursor

**Figure 3:**
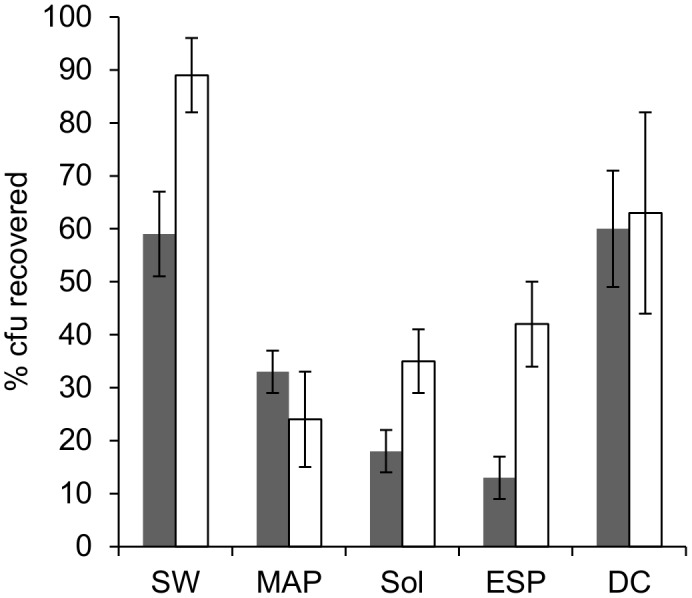
Survival of *B. safensis* spores in uncured polymer precursors (gray bars) relative to the initial inoculum (10^7^ spores/mL, dilution in aqueous diluent), and growth of 10^2^ cfu of *B. safensis* in the presence of uncured polymer precursors on nutrient agar (white bars) relative to the population of *B. safensis* grown without precursors.

The possible sporicidal effect of the uncured polymer precursors was tested by inoculating Scotch-Weld 2216 B/A as described in Materials and Methods. Cultivable spore recovery directly after inoculation was 59% (**Fig. 3**). The number of retrievable cultivable spores remained stable over the incubation period of 60 min (not shown). An inhibitory effect of the solvent (2-propanol) used for dilution of the material after the incubation period had been experimentally excluded in preliminary experiments.

### Application of Test Model II for Scotch-Weld 2216 B/A: Recovery Efficiency of Inoculated Spores from Polymer Precursors in Presence of Organic Solvents

Colony formation of activated spores in the pour-plate medium was only slightly inhibited by the presence of Scotch-Weld 2216 B/A and solvent residues. The recovery efficiency of spores amounted to 89% (**Fig. 3**), which was higher, but in the same order of magnitude as survival in the precursors. This discrepancy was due to the difference in experimental procedure to determine survival and recovery, respectively. In survival determination, a high inoculum was used, which was consecutively diluted before plating, which may have caused some loss of spores. The recovery efficiency (ratio of recovered cfu in nutrient medium in presence and absence of polymers) was used to correct the value for total intrinsic bioburden.

### Application of Test Model II for Scotch-Weld 2216 B/A: Encapsulated Viable Intrinsic Bioburden of Uninoculated Polymer Precursors

A recovery efficiency of >10% was arbitrarily specified as acceptance criterion for extraction of natural encapsulated bioburden from polymer precursors because the quantity of the material studied was high (87 g or 66 cm^3^ for Scotch-Weld 2216 B/A; **Table 3**; investigated in 80 separate samples). In the total amount of Scotch-Weld 2216 B/A investigated, 0 cfu were found on the agar plates (**Table 3**). Thus, contamination of Scotch-Weld 2216 B/A with spores cultivable under the given conditions was calculated to be below 0.1 cfu/cm^3^ (**Table 3**).

**Table 3:** Contamination of the polymer materials with bacterial endospores determined by dilution in solvent and cultivation (corrected values calculated with recovery efficiency).

Material	Amount of material investigated (cm^3^)	Total spore-forming colonies detected (cfu)	Encapsulated bioburden (cfu/cm^3^), corrected
Scotch-Weld 2216 B/A	66	0	<0.1
MAP SG121FD (batch 1/batch 2)	13/50	5/0	2.4/<0.3
Solithane 113	81	1	0.1
ESP 495	45	2	0.3
DC 93–500	59	0	<0.1

### Application of Test Model II for Scotch-Weld 2216 B/A: Molecular Determination of Contaminants in Polymer Precursors

Quantitative PCR (qPCR) was applied to estimate the total natural bioburden of the polymer materials, including both dead and potentially viable cells. However, DNA extraction from polymer precursors proved very difficult. Although Scotch-Weld 2216 B/A could be dissolved properly in 2-propanol, remaining residues and clay particles bound biomaterial effectively, so that a separation of polymer and biomolecules (incl. cells and DNA) was impossible. This resulted in a very poor recovery of spore signatures, which were added as positive control (<0.1% DNA extraction efficiency).

### Application of Test Model II for Scotch-Weld 2216 B/A: Contribution of the Filler Material Kaolin to the Overall Bioburden Determined by Molecular Methods and Cultivation

**Figure 4:**
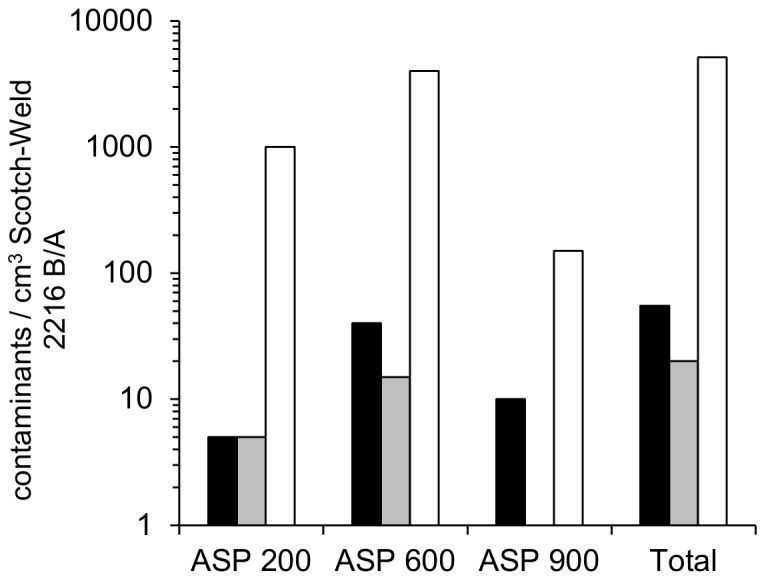
Microbial contamination per cm^3^ Scotch-Weld 2216 B/A contributed by filler material kaolin (3 grain sizes ASP 200, 600 and 900), as determined by cultivation (cfu  =  without heat shock, black bars; cultivable spores  =  heat-shock-survivors, gray bars) and by qPCR (total contaminants (*rrnB-16S* gene copies, white bars). Standard deviations are not shown as the data is only meant to give a broad orientation. For a statistically sound analysis a much higher sample number would need to be investigated.

Scotch-Weld 2216 B/A contains about 30% (w/w) of kaolin as a mineral filler material (see fiber-like inclusions in **Fig. 1C**). Bioburden of kaolin was determined separately, since dissolution by solvents was impossible. In addition, due to its natural origin, this filler was suspected to be a source of elevated microbial contamination. Kaolin ASP 200, 600 and 900 (3 different grain sizes) were separately tested for microbial contamination by cultivation on three different media as well as by DNA extraction and qPCR. As kaolin could be suspended in water, DNA extraction efficiency was higher than for the final polymeric material Scotch-Weld 2216 B/A. The relevance of kaolin as contamination source for Scotch-Weld 2216 B/A, in consideration of the contribution of the filler to the weight of cured Scotch-Weld 2216 B/A and the density of Scotch-Weld 2216 B/A (1.32 g/cm^3^), was determined by the different methods as summarized in **Fig. 4**. Microbial contamination introduced into Scotch-Weld 2216 B/A by kaolin was estimated by qPCR to be in the range of 10^3^ (potential vegetative cells or spores)/cm^3^. Only a fraction of this number (∼1%) could be recovered by cultivation on agar. It should be noted, that the calculated number of microbial contaminants based on molecular methods can only be a rough estimate as the number of *rrnB-16S* gene copies per genome and cell is unknown for unidentified microbes. Furthermore, this method also detects DNA from dead cells, permitting no quantification of viable cells.

### Application of Test Model II for other Polymers

In addition to Scotch-Weld 2216 B/A, four other space craft relevant polymers, of different type and composition were also investigated: SG121FD a silicone coating; Solithane 113, a urethane resin; ESP 495 a silicone adhesive; and Dow Corning 93–500 (DC 93–500) a silicone encapsulant (**Table 1**). Bioburden determination of uncured polymer precursors was performed following Test Model II.

#### Application of test model II for other polymers: survival of *B. safensis* spores in uncured polymer precursors

**Figure 5:**
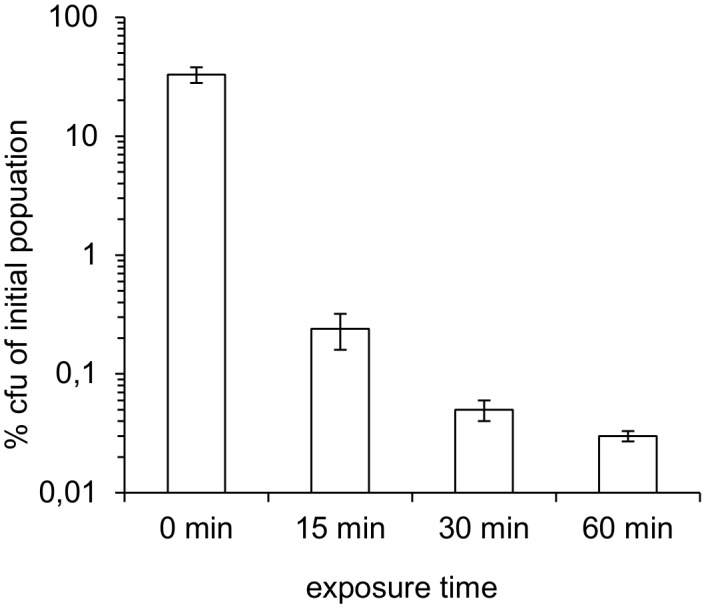
Survival of *B. safensis* spores in uncured MAP SG121FD during 0–60 min of incubation.

The possible sporicidal effect of the polymer precursors was tested as described in Materials and Methods. Recovery directly after inoculation was >10% for all tested polymer materials (**Fig. 3**), ranging from 13% in ESP 495 to 60% for DC 93–500. The number of cultivable spores remained stable over the incubation period three of the selected materials, whereas in the uncured silicone coating MAP SG121FD, a reduction of cultivable spores (to 0.03% of the initial inoculum) within 60 min of exposure indicated a pronounced sporicidal effect (**Fig. 5**). An inhibitory effect of the solvent (SG121FD thinner) used for dissolution of MAP SG121FD after the incubation period could be experimentally excluded.

### Application of Test Model II for other Polymers: Recovery Efficiency of Spores from Polymer Precursors in Presence of Organic Solvents

Colony numbers of *B. safensis* spores were slightly reduced by the presence of polymers and solvents in the solid growth medium in all cases, but colony counts exceeded 10% (**Fig. 3**), ranging from 24% in MAP SG121FD to 63% in DC 93–500. As noted before, higher recovery efficiencies compared to survival ratios may be due to loss of spores during the serial dilutions performed for survival determination. In general, survival and recovery was in the same order of magnitude.

### Application of Test Model II for other Polymers: Encapsulated Cultivable Bioburden of Uninoculated Polymer Precursors

The number of colonies recovered from the unspiked polymer precursors by solvent dilution and incubation in solid nutrient medium was very low in all cases. In 87 g (81 cm^3^) Solithane 113, 1 cfu was recovered, giving a corrected bioburden of 0.1 cfu/cm^3^. The detected colony consisted of gram-positive, rod-shaped cells with visible endospores. One colony was also recovered from 64 g (59 cm^3^) DC 93–500. However, as the isolate did not survive heat shock treatment when suspended in solvent, it was very likely a secondary contamination of vegetative organisms. Bioburden of DC 93–500 was determined to be below 0.1 cfu/cm^3^. Investigation of 48 g (45 cm^3^) ESP 495 yielded 2 cfu of spore-forming organisms, one of which appeared under elevated temperature (55–60°C) incubation. Although 4 more colonies were recovered, those consisted of vegetative organisms, sensitive to the extraction procedure and were considered secondary contaminations. Thus, the corrected intrinsic bioburden of ESP 495 amounted to 0.3 cfu/cm^3^. In MAP SG121FD coating, results varied for the two analyzed batches. 5 cfu were recovered after incubation under mesophilic conditions in the first batch of SG121FD (18 g or 13 cm^3^ investigated). Gram-staining of these colonies showed gram-positive, rod-shaped cells, indicating spore-forming organisms. No colony was detected in the second batch of SG121FD, of which a higher amount was analyzed (71 g or 50 cm^3^). It should be taken into consideration that cultivability of *B. safensis* spores was rapidly reduced within 60 min of exposure in SG121FD (**Fig. 5**). The spore-forming organisms, which were detected in one of the batches of SG121FD could have been more resistant to the inhibitory effects of the silicone coating than *B. safensis*.

### Application of Test Model II for other Polymers: Molecular Determination of Contaminants in Uninoculated Polymer Precursors

**Figure 6:**
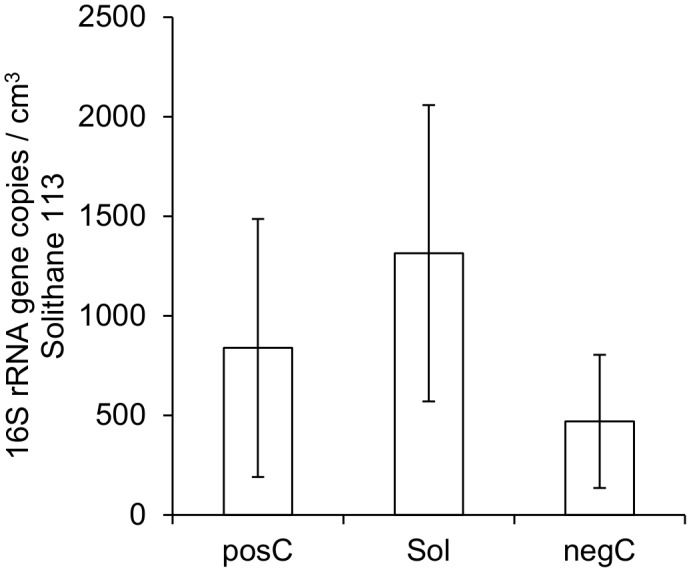
Mean molecular biocontamination of Solithane 113 determined by qPCR.

Solithane 113 was selected in addition to Scotch-Weld 2216 B/A to attempt the application of molecular methods. When attempting to extract DNA from Solithane 113, similar obstacles arose as for Scotch-Weld 2216 B/A. Although diluted in ethanol and using an adapted DNA extraction protocol, uncharacterized polymer residues masked targeted biomolecules for subsequent molecular analyses. 2×10^6^ spores were added as a positive control, resulting in the detection of approx. 10^4^ *rrnB-16S* gene copies via qPCR (less than 0.5% extraction efficiency; in case the average spore contains more than a single gene copy, the extraction efficiency would be even lower, as expected. In addition, polymer components in the DNA suspension caused interference with the qPCR signal, which had to be adjusted by application of a correction factor. Nevertheless, Solithane 113 samples revealed a higher contamination with *rrnB-16S* genes than positive controls (**Fig. 6**). Since the recovery efficiency for the total natural bioburden from Solithane 113 is unknown, the true bioburden cannot be estimated. However, a minimum contamination of approx. 4×10^3^ gene copies per 5 g material (500/g or 535/cm^3^) can be envisaged.

## Discussion

Planetary protection ensures the validity of future life detection missions on foreign celestial bodies by biological control of spacecraft and its assembling environment in clean room facilities [42]. Since the Viking mission era, spacecraft surface associated bacterial spore burden has been assessed by standard cultivation protocols [43], [44], [45], [46]. Recent approaches were based again on cultivation-level [47], or applied new promising molecular methods, since cultivation is limited to standard laboratory conditions and bears the risk to ignore the majority of microorganisms in a sample [48], [7], [49], [50]. The knowledge about polymer encapsulated bioburden, however, remains sparse, most likely due to difficulty to retrieve the organisms from cured polymers. Milling of cured polymers was considered impractical as it involves mechanical shear forces and local heating, which are very difficult to control and their effect on encapsulated cells or spores is unknown [51], [52]. Comparable hurdles have been described by [14] and [26] during recovery and detection of encapsulated bioburden in silicone or Plexiglas and had to be overcome by adapted extraction and permeabilization protocols. While the polymers used in the aforementioned studies were selected specifically to study encapsulation models, the aim of the present study was to find test models that can be used to characterize the intrinsic bioburden of spacecraft-relevant polymers. The test models developed for Scotch-Weld 2216 B/A, and applied to ESP 495, DC 93–500, MAP SG121FD and Solithane 113 can be used for varied test strategies to characterize and estimate the intrinsic bioburden of polymers. The models lend themselves to investigate not only the most frequently used polymers for spacecraft but also other polymers used in food and pharmaceutical industry or medical devices.

We first developed a surface-embedding model that allowed easy access to the inoculated spores for assessment of the effect of polymerization on encapsulated *B. safensis* spores in Scotch-Weld 2216 B/A as an exemplary polymer. Without any disruptive extraction from the polymer, embedding effects could be studied with a minimum of preparation artifacts. Fluorescently labelled spores, which could be detected to a depth of 3 µm, showed spore coat integrity and respiratory metabolism in presence of provided nutrients and some of them were able to germinate and form colonies. Thus, it cannot be assumed that the polymerization process is damaging to spores, although this result is expected to vary with different polymeric materials as well as with spore structure. For materials that lend themselves to application of the described or similar methods, our developed encapsulation model in combination with molecular staining techniques can be a valuable tool to assess how the germination ability of spores is affected by polymerization and gives additional information about embedded spores to the approach with Alexa-FISH presented by Mohapatra and La Duc [26].

Although the model presented by Mohapatra and LaDuc was pioneering work and yielded interesting data on spiked spores, it could not be applied to quantitatively estimate the intrinsic bioburden of polymers. The intrinsic bioburden of polymers is expected to vary for a number of reasons: i) they are composed of widely different materials, ii) some include materials of natural origin (e.g. fillers), iii) manufacturing conditions for the polymers are diverse and iv) uncured materials may have different effects on microorganisms. For estimation of intrinsic bioburden in a broad range of polymers a method for lot-specific quantitative estimation of the intrinsic bioburden was sought. We developed a test model that combines cultivation-based and molecular methods for quantitative bioburden determination. Since biocompatible disruption or dissolution of the cured polymers was found to be impossible in this study, uncured polymer precursors were diluted in solvents before polymerization. This was considered the most conservative and also the least sporicidal approach. Inoculated endospores (shown to be insensitive to the employed diluents) could be recovered with reasonable effectiveness. Hence, the strategy applied herein was shown to be applicable to determine the maximum number of cultivable intrinsic spores in a wide range of polymers.

A low contamination with encapsulated cultivable endospores was found for all investigated polymer materials, in the range of <0.1 spores/cm^3^ to a maximum of 2.4 spores/cm^3^ in one batch of MAP SG121FD. SG121FD was the only tested material, for which a sporicidal effect on inoculated model spores was observed over incubation periods of ≤60 min. Thus, the risk of germinable spore contamination for this material was considered to be low.

The bioburden of polymer precursors is specific for each material and can vary from batch to batch (as shown for MAP SG121FD). For practical application a number of batches would have to be tested, and any change in the manufacturer or the manufacturing process of the polymer material would necessitate re-evaluation of its bioburden.

As for previous attempts of bioburden evaluation [43], [44], [45], the limitation of this method was the use of cultivation-based detection methods, which are known to detect only a fraction of the total number of viable organisms present in a sample of natural origin [53], [54], [55]. However, if the recovered spores are unable to germinate under optimal conditions, the probability for germination and propagation on a possibly hostile planetary surface might be considered minute, even if phenomena like superdormant spores are regarded [56].

Nevertheless, for planetary protection purposes, dead and not-yet-culturable organisms need to be considered as well, since they might interfere with life or biomarker detection methods. Therefore, determinations of the molecular bioburden were considered an important complement to cultivation-based assays. However, DNA extraction from the polymer precursors of Scotch-Weld 2216 B/A proved difficult, because DNA was bound by the polymer particles diluted in solvent as described to be the case for silicones, too [14]. Hence, promising high throughput omics technologies (e.g. transcriptomics and proteomics) could not be applied beyond our qPCR and microscopy based approaches. Due to the low extraction efficiency, quantitative data could not be obtained for Scotch-Weld 2216 B/A, but for its filler kaolin, which amounted to ∼10^3^ cells/cm^3^ (estimation from *rrnB-16S* gene copies determined via qPCR). This amount exceeded by far the number of microorganisms that could be detected by cultivation in the same material. However, a general conclusion for bioburden of this filler is difficult to draw, since kaolin is a natural resource originating in clay mines, which are exposed to various environmental influences, and harbor a remarkable diversity of extremophiles like iron oxidizing and reducing bacteria (dark iron oxides and sulfides are major discoloring impurities of white kaolin [57]), and acidophilic archaea and bacteria (sulfidic mine waters [58]). Additionally, mining and processing of kaolin is not performed under sterile conditions and may vary for different manufacturers. Nevertheless, this result was in accordance with our expectations, as molecular methods include DNA from dead organisms, as well as from viable and viable-but-nonculturable organisms. The high numbers of potentially viable microbial contaminants (also found in previous studies by other authors, e.g. [59], indicate that fillers of polymers should not be dismissed in studies of encapsulated bioburden.

### Recommendations for Future Studies

We recommend the application of the presented methods to other materials for which an evaluation of encapsulated bioburden is important. Such materials may be other adhesives, coatings (silicone) and bulk polymers (polyurethane, silicone and polyimide), honeycomb panels, heat shields (Norcoat Liege cork) and components like wires and connectors [60]. Since these materials are quite diverse, in each case a risk analysis is recommended to decide, if viable bioburden is expected to be present at all. For composite materials like wires and connectors it is important to investigate whether bioburden resides in the polymeric insulation materials, in between the insulating layers, or on the wires themselves. The testing strategy will have to be adjusted accordingly, and a special set of methods for evaluating its bioburden will have to be posed.

The method of choice for components where dissolvable precursors are available is Test Model II, resulting in the most conservative evaluation for embedded spores. A suitable surface embedding model where added microorganisms are easy to detect and analyse in various depths of embedding as presented in our Test Model I could be helpful for evaluating polymerization effects on the viability of other microbes.

Presented physiological markers with fluorescent activity are quick and applicable given that the material itself does not show severe auto-fluorescence. If the material has to be considered a potential contamination risk and especially if auto-fluorescence is preexisting [26], a separate analysis (e.g. qPCR) of certain components as performed for the kaolin filler might be appropriate. As revealed by our study, separate sterilization of such natural components would be a good alternative in order to reduce the viable encapsulated bioburden. In addition, it might also be important to phylogenetically, metabolically and physiologically characterize the detected bioburden to estimate its risk for planetary protection.

## Conclusion

With polymeric components of spacecraft sent from earth to other celestial bodies, but also with the ubiquitous use of polymer materials in food and pharmaceutical industry, the question of their possible internal bioburden has been raised. Here, we have investigated methods that can be applied to estimate the number of persistent microorganisms encapsulated in different polymeric materials. The methods, which were presented as a composite strategy by use of viability staining of embedded microorganisms, classic cultivation procedures, and molecular detection techniques to evaluate encapsulated bioburden of spacecraft model hardware, are not only of interest for the research field of planetary protection. For food [61] and pharmaceutical industry [62], [63], [64] and clinical equipment [65], an evaluation of cleanliness and sterility is also of great importance, since these fields are confronted by new threats like multi-resistant bacteria and pathogens [66]. Bioburden embedded in polymeric materials is of interest wherever plastic materials are used. Survival of bacterial endospores embedded in such materials is of significance when the huge amount of plastic materials is considered, that is rotting in dumps or littering the surface of the earth and the oceans [67]. These materials do not only leach chemicals but may also harbor microorganisms that get released and proliferate.

Our protocols proved feasible for five typical spacecraft polymers, and we can state that the overall cultivable endospore burden inside the analyzed polymers was low with <0.5 spores/cm^3^ for most of the investigated materials. Furthermore, beyond cultivation and molecular-based methods, this study shows the potential of physiological staining methods like AF488, PMA and CTC to evaluate embedded spores in polymers. The combination of these dyes demonstrated for the first time the capability of spores to withstand mechanical forces during embedding in and curing of a polymer.
